# Epigenetic Analysis in Human Neurons: Considerations for Disease Modeling in PD

**DOI:** 10.3389/fnins.2019.00276

**Published:** 2019-04-05

**Authors:** Laura de Boni, Ullrich Wüllner

**Affiliations:** ^1^Dementia Research Institute, University College London, London, United Kingdom; ^2^Department of Neurology, University Hospital Bonn, German Center for Neurologic Diseases, Bonn, Germany

**Keywords:** stem cells, iPSC, neurons, human, epigenetics, Parkinson’s disease

## Abstract

Parkinson’s disease (PD) is the second most common neurodegenerative disorder next to Alzheimer’s disease. Most PD cases are considered to be sporadic and despite considerable scientific effort, the underlying cause(s) still remain(s) enigmatic. In particular, it is unknown to which extent epigenetic alterations contribute to the pathophysiology of this devastating disorder. This is partly due to the fact that appropriate PD models are not yet available. Moreover, epigenetic patterns and mechanisms are species specific and murine systems reflect only a few of the idiosyncrasies of human neurons. For several years now, patient-specific stem cell-derived neural and non-neural cells have been employed to overcome this limitation allowing the analysis and establishment of humanized disease models for PD. Thus, several studies tried to dissect epigenetic alterations such as aberrant DNA methylation or microRNA patterns using lund human mesencephalic cell lines or neurons derived from (patient-specific) induced pluripotent stem cells. These studies demonstrate that human neurons have the potential to be used as model systems for the study of epigenetic modifications in PD such as characterizing epigenetic changes, correlating epigenetic changes to gene expression alterations and hopefully using these insights for the development of novel therapeutics. However, more research is required to define the epigenetic (age-associated) landscape of human *in vitro* neurons and compare these to native neurons before they can be established as suitable models for epigenetic studies in PD. In this review, we summarize the knowledge about epigenetic studies performed on human neuronal PD models, and we discuss advantages and current limitations of these (stem cell-derived) neuronal models for the study of epigenetic alterations in PD.

## Introduction

Parkinson’s disease (PD) is the second most common neurodegenerative disorder affecting 1–2% of the population aged >65 years (Tanner and Goldman, 1996). The vast majority (>90%) of PD cases are non-familial and are considered to be sporadic (sPD) (Deng et al., 2018). Despite considerable scientific effort in the last decades, the underlying cause for sPD still remains enigmatic. Not a single cause, but rather several mechanisms appear to underlie the individual susceptibility of sPD (Marras and Lang, 2013). Indeed, several cluster analysis of clinical features identified subtypes in PD pointing to different clinic phenotypes which may correspond to different gene-environment interactions and individual susceptibility (Erro et al., 2013).

**FIGURE 1:**
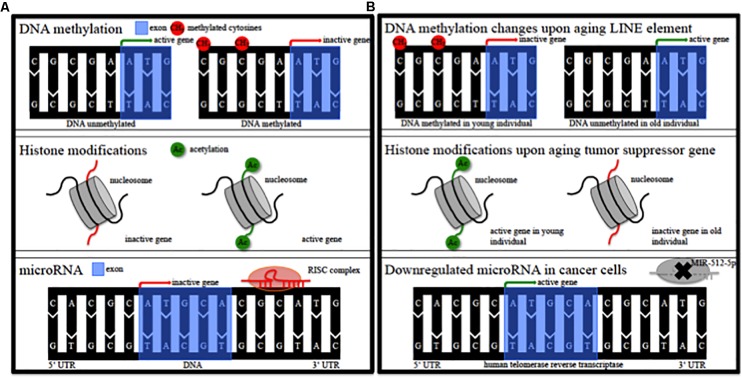
Epigenetic modifications. **(A)** Schematic of epigenetic modifications comprising DNA methylation, histone modifications, and microRNA. RISC, RNA-induced silencing complex. **(B)** Schematic of possible epigenetic modifications during aging and in cancer tissue. LINE, long interspersed nuclear element; MIR, microRNA.

Epigenetic alterations of genes could contribute to the pathophysiology of this individual susceptibility (Landgrave-Gomez et al., 2015). Epigenetic modifications comprise DNA methylation, modification of histones, non-coding RNA regulation, RNA editing and nucleosome remodeling/positioning (Figure 1A) (Portela and Esteller, 2010; Wen et al., 2016; Qureshi and Mehler, 2018). These epigenetic modifications regulate gene expression without impact on the DNA sequence itself (Portela and Esteller, 2010; Wen et al., 2016; Qureshi and Mehler, 2018). Epigenetic modifications regulate fundamental cellular processes (e.g., gene transcription, X-chromosome inactivation) and fill the gap between environmental and age-associated gene expression (Wen et al., 2016; Qureshi and Mehler, 2018). They are dynamic, cell specific, display interindividual variability and can also occur in non-dividing cells such as neurons (Fraga et al., 2005; Feng et al., 2007; Fraga and Esteller, 2007). DNA methylation in particular modulates gene expression at promoter or intragenic loci and importantly also via modification of (regulatory), non-coding DNA-sequences (Wullner et al., 2016). Epigenetic patterns change in cancer tissue (Figure 1B) and upon aging (Figure 1B) in various organs and the analysis of DNA methylation levels can provide an independent indicator of aging (i.e., the “epigenetic clock”) (Horvath, 2013). Mutations of epigenetic regulatory genes in neurological diseases include DNA methyltransferases [DNMT1, hereditary sensory and autonomic neuropathy type 1 (Klein et al., 2011)], histone transferases [CREB binding protein, Rubinstein-Taybi syndrome (Petrif et al., 1995)], or Methyl-CpG-binding proteins [MeCP2, Rett syndrome (Amir et al., 1999)]. In addition, mutations in genes can lead to secondary epigenetic modifications. For example, CGG expansion mutations in the fragile X mental retardation 1 (*FMR1*) gene result in hypermethylation of the 5′ UTR of *FMR1* in patients, leading to decreased *FMR1* protein levels (Liu et al., 2018). Furthermore, different groups of cortical genes undergo profound and characteristic age-dependent DNA methylation alterations (Siegmund et al., 2007). In general, aging is associated with a global decrease in DNA methylation levels and aged monozygotic twins display significant epigenetic differences, a process referred to as epigenetic drift (Fraga et al., 2005; Teschendorff et al., 2013). Profound cortical DNA methylation differences have been reported in monozygotic twins discordant for Alzheimer’s disease (Mastroeni et al., 2009).

Elevated expression of α-synuclein, an important key player in PD, has been linked to the development of PD (Singleton et al., 2003; Ibáñez et al., 2004). Thus, studies tried to identify DNA methylation alterations of the α-synuclein gene (*SNCA*) (Spillantini et al., 1997; Jowaed et al., 2010; Matsumoto et al., 2010; de Boni et al., 2011). However, these findings remain controversial as some studies identified DNA methylation alterations of *SNCA* in post-mortem brain tissue from PD patients (Jowaed et al., 2010; Matsumoto et al., 2010; Desplats et al., 2011), whereas others could not replicate these findings (de Boni et al., 2011; Guhathakurta et al., 2017).

The in-depth analysis of presumed epigenetic alterations in sPD is hampered by the lack of appropriate sPD models. Several phenomena have to be considered:

(I) Neurodegenerative diseases (NDDs) do not have any equivalents in the animal kingdom and are specific to humans except for some rare examples in aged dolphins or baboons which are not dispensable for research (Lowenstine et al., 2016; Gunn-Moore et al., 2018).
(II) Epigenetics both in terms of structure (DNA methylation), histone code and functional interplay of the various enzymes are species specific (Mendizabal et al., 2014; Lowdon et al., 2016). Thus, murine (or other) model systems are not representative for human conditions.
(III) NDD occur as non-familial diseases in the vast majority of cases. A limited number of autosomal (and recessive) mutations have been identified. Exploring these inherited forms in transgenic cell culture and animal models to transfer the results to the sporadic cases has been the mainstream research strategy for the past 30 years. However, at least in PD, the discovery and exploitation has not resulted in a decisive clue to the etiology of the non-familial PD cases. Clinical trials of compounds identified in several transgenic and toxin models have failed (Olanow et al., 2008; Löhle and Reichmann, 2010).
(IV) More appropriate disease models, i.e., living human neurons have become available only recently.

## Challenges Choosing the Right Model to Study Epigenetic Alterations in sPd

To date, there is no decisive clue to the aetiology of sPD and consequently no causal treatment. This lack of knowledge represents a tremendous burden for the patients, their families and caregivers. PD treatment relies on symptomatic therapies, neither slowing nor halting the process of neurodegeneration. Further, a biomarker identifying PD patients before the onset of motor symptoms, i.e., before pronounced neurodegeneration, is lacking. Thus, the development of novel therapeutics is challenged and current clinical trials (e.g., Isradipine, phase III) try to focus on early PD patients not requiring a symptomatic therapy (Biglan et al., 2017). Moreover, current animal models do not fully reflect the underlying pathology and hallmarks of PD and despite huge efforts, could not elucidate the underlying causal pathology towards sPD. The analyses of native brain tissue is thus necessary, but limited to post-mortem tissue, which is not suited for mechanistic studies. Studies on epigenetic alterations using native bulk post-mortem brain tissue generated conflicting results (Jowaed et al., 2010; de Boni et al., 2011).

Common cellular human models comprise the neuroblastoma cell line SK-N-SH and its subclone SH-SY5Y. Indeed, these cancer cells helped to identify important pathways and mechanisms in PD, e.g., mitochondrial dysfunction or impaired autophagy (Zeng et al., 2018). Upon treatment of SK-N-SH cells with the DNMT inhibitor 5-Azacytidine, DNA demethylation of the *SNCA* intron 1 was associated with an increase in *SNCA* mRNA and α-synuclein protein expression (Jowaed et al., 2010). To date, these findings could not be replicated in human neurons due to the inaccessibility of living disease-affected cells. It remains to be determined whether alteration in *cis* or yet undetermined exogenous factors underly epigenetic modifications of the *SNCA* gene.

Epigenetic variation plays an important role in evolution (Richards, 2008; Mendizabal et al., 2014). Many animal genomes (mostly vertebrates) are depleted of CpG dinucleotides and genes associated with neurological functions exhibit human-specific patterns of DNA methylation (Mendizabal et al., 2014). Whole-genome DNA methylation analysis of prefrontal cortical tissue of humans and chimpanzees revealed extensive species-specific variation associated with strong gene expression changes (Zeng et al., 2012). Moreover, it has already been demonstrated on a single-cell level that mouse and human embryos are characterized by distinct differences in DNA methylation and histone modification patterns (Chavez et al., 2014).

To date, there is no decisive clue to the etiology of sPD and consequently no causal treatment. This lack of knowledge represents a tremendous burden for the patients, their families and caregivers. PD treatment relies on symptomatic therapies, neither slowing nor halting the process of neurodegeneration. Further, the lack of a biomarker which would identify PD patients before the onset of motor symptoms, i.e., before pronounced neurodegeneration has occurred. Current clinical trials (e.g., Isradipine, phase III) try to focus on early PD patients not requiring a symptomatic therapy (Biglan et al., 2017). Isradipine was identified in epidemiological studies and has shown “rejuvenating” effects *in vitro* and *in vivo* models. It is supposed that Isradipine blocks L-type Ca(v)1.3 Ca^2+^ channels maintaining their rhythmic pacemaking which in turn results in decreased vulnerability of the neuron to cell stressors (Chan et al., 2007). It remains to be determined whether the current clinical trial, in contrast to the previous trials of compounds identified in transgenic and toxin models will be successful (Olanow et al., 2008; Löhle and Reichmann, 2010).

More appropriate disease models, i.e., living human neurons have become available only recently and could be used to study epigenetic modifications in PD. Two immortalized human male fetal neural precursor lines were developed from cortex (CX) and the ventral mesencephalon (VM) carrying the c-myc or v-myc oncogene, respectively. These so-called ReNcell CX and VM express typical markers for neural precursor cells and upon differentiation, ReNcell VM showed positive staining for tyrosine hydroxylase (Donato et al., 2007). Another mesencephalic human cell line was derived from 8-week-old female human ventral mesencephalic tissue and a subclone, the lund human mesencephalic (LUHMES) cell line, can be differentiated into morphologically and biochemically mature dopamine-like neurons (Lotharius et al., 2005). The advantage of these cell lines is that they consist of a quite homogenous population, have the ability to be cultured and expanded long-term, have the capacity to be differentiated into more mature post-mitotic dopaminergic phenotypes and are suitable for to novel genetic editing tools such as the Clustered Regularly Interspaced Short Palindromic Repeats (CRISPR)/Cas system.

Human stem cell-derived neurons can be generated from embryonic stem cells (ESC), induced pluripotent stem cells (iPSCs) or tissue-specific (stem) cells. ESC are obtained from the inner cell mass of the blastocyst and are pluripotent (Thomson et al., 1998; Dhara and Stice, 2008). Diverse protocols were utilized to directly differentiate ESC into neural (and glial) cells under defined culture conditions using different factors, signaling molecules, supplements, and additives (Dhara and Stice, 2008). Differentiated cells such as fibroblasts can be reprogrammed by defined factors to iPSC resetting an embryonic state (Nakagawa et al., 2008; Eguchi and Kuboki, 2016). IPSC can be derived from patients and controls allowing the study of disease- and non-affected defined neuronal populations and other cellular subtypes. Tissue specific cells such as dermal (Joannides et al., 2004) or mesenchymal stem cells (Singh et al., 2017) can also be differentiated into neural cells. Direct conversion – omitting a pluripotent intermediate state – of somatic cells into so-called induced neurons (iN) exhibiting typical neuronal and differentiation properties is achieved by defined transcription factors (Ladewig et al., 2012; Prasad et al., 2016; Xu et al., 2017). Regarding PD, most attempts have focused on generating *in vitro* DAn to study disease-related mechanisms as cardinal motor manifestations of PD refer to the progressive loss of DAn. Stem cell-derived DAn were generated by many research groups using quite different approaches and culture conditions. Most protocols are based on activation of the important Sonic Hedgehog and WNT signaling pathway in combination with floor plate based and feeder cell protocols or other small molecules and together with transcription factors required for proper DAn specification such as FOXA2, LMX1A, or Oct4 (Chen et al., 2009; Cooper et al., 2010; Deleidi et al., 2011; Kriks et al., 2011; Kirkeby et al., 2012; Arenas et al., 2015; Fernandez-Santiago et al., 2015; Fedele et al., 2017; Qiu et al., 2017). Other protocols used retinoic acid (Tsai et al., 2015), Vitamin C (Wulansari et al., 2017), supplementation with steroids (Agbay et al., 2018), FGF20 (Correia et al., 2007), or plating in Lam-111 (Kirkeby et al., 2017) to increase the yield of DAn. Indeed, the attempt to study a defined and especially vulnerable neuronal population is imperative. However, it should be noted that the marker expression and efficiency generating DAn vary highly depending on the applied protocol (Xu et al., 2017). Thus, observed PD related phenotypes (i.e., mitochondrial dysfunction, α-synuclein accumulation, synaptic dysfunction) might not be solely linked to DAn. PD related phenotypes have nevertheless been detected in iPSC-derived DAn and neurons from *PARK2/Parkin* (Imaizumi et al., 2012; Ren et al., 2015; Chung et al., 2016), *PINK1* patients (Chung et al., 2016), PD patients carrying glucocerebrosidase (*GBA*) mutations (Schondorf et al., 2014; Woodard et al., 2014; Aflaki et al., 2016; Fernandes et al., 2016; Yang et al., 2017), patients harboring the *SNCA* triplication mutation (Byers et al., 2011; Flierl et al., 2014; Oliveira et al., 2015), and in iPSC-derived DAn and sensory neurons from patients carrying leucine-rich repeat kinase 2 (*LRRK2*) mutations (Nguyen et al., 2011; Bahnassawy et al., 2013; Schwab and Ebert, 2015; Hsieh et al., 2016; Lopez de Maturana et al., 2016; Sandor et al., 2017; Schwab et al., 2017). Several studies have already tried to decipher mechanistic processes and tested drugs using iPSC-derived DAn: DAn from *SNCA* triplication carriers secreted high levels of α-synuclein which was taken up by neighboring neurons (Reyes et al., 2015). FACS-sorted post-mortem DAn from *LRRK2*-G2019S carriers displayed a distinct transcriptome compared to post-mortem DAn from non-affected individuals and these DAn also expressed a distinct set of genes compared to control DAn (Sandor et al., 2017). Clioquinol, a compound known to alleviate dopamine neuron loss, rescued these pathogenic expression changes (Sandor et al., 2017). The macrolide compound rapamycine and the PGC1α-transcription factor inducing disaccharide trehalose protected against mitochondrial neurotoxicity in iPSC-derived DAn exhibiting the Parkin Q311X mutation (Siddiqui et al., 2015). High-throughput screening identified the compounds NCGC607 and NCGC00188758 and the chaperone ambroxol, which restore glucocerebrosidase activity and rescue PD-related phenotypes in DAn from *GBA* carriers (Aflaki et al., 2016; Mazzulli et al., 2016; Yang et al., 2017). The effect of NCGC00188758 was confirmed in iPSC-derived DAn of *SNCA* triplication, *SNCA* A53T, sPD and PARK9 patients (Mazzulli et al., 2016). These aforementioned studies however bear only little impact regarding the pathophysiological processes of sPD. It remains to be determined whether human (patient-specific) neurons might also be used to generate useful models to identify novel pathophysiological processes to such as epigenetic modifications in PD.

## Epigenetic Studies in Human Neurons

### Epigenetic Studies Using Immortalized Human Fetal-Derived Neurons

#### HDACi Rescue WT α-Synuclein-Induced DNA Damage in LUHMES Cells

Paiva et al. (2017) demonstrated transcriptional deregulation (e.g., downregulation of the dopamine receptor; DAT) in α-synuclein wild type (WT) and A30P lentiviral transfected LUHMES cells differentiated for 8 days (Table 1). RNAseq demonstrated a downregulation of DNA repair genes due to the overexpression of WT and A30P α-synuclein. The expression of WT α-synuclein was associated with a significant reduction in the levels of acetylated Histone 3 and treatment of cells using the HDACi sodium butyrate could ameliorate the observed phenotype. These results were confirmed in LUHMES cells treated with 1-methyl-4-phenylpyridinium (MPP^+^).

**Table 1:** Epigenetic studies in immortalized human fetal-derived neurons.

Study	Cell source	Neuronal differentiation	Modification of cell lines	Methods	Results
Chaudhuri et al., 2015	ReNcells VM	DAn, 2 weeks old	Lentiviral transfection of pre-MIR, anti-MIR and siRNA	Different assays (e.g., Glucose/Lactic acid, Pyruvatkinase, cell viability, apoptosis), qRT-PCR, WB, measurement of neurite length	MIR-7 is cytoprotective and exhibits anti-neurotoxic effects
Paiva et al., 2017	LUHMES	DAn, 8 days old	Lentiviral transfection of IRES-GFP control, WT α-Syn-IRES-GFP, A30P α-Syn-IRES-GFP	RNA-Seq, WB, qRT-PCR, single cell gel electrophoresis, ROS detection assay, ToxiLight bioassay kit	HDACi rescue WT α-synuclein DNA damage
Pierce et al., 2018	LUHMES	DAn, 6–7 days old		Chip-Seq, sequencing of directional mRNA-seq libraries	PD risk SNPs are enriched in enhancers in DAn
Oh et al., 2018	ReNcells VM	DAn, 10 days old	shRNA DJ1 KO, lentiviral transfection of MIR-221	Affymetrix 2.0 ST RNA, computational methods, qRT-PCR, cell viability and cell death assays, WB, Immunocytochemistry	MIR-221 expression is modulated by WT DJ1 to execute cytoprotective effects
Choi et al., 2018	ReNcells VM	DAn, 7 days old	Transfection of Lentiviral MIR-7 and adenoviral α-synuclein	WB, immunocytochemistry	Overexpression of MIR-7 ameliorates α-synuclein-associated phenotypes

*VM, ventral mesencephalon; DAn, dopaminergic neurons; MIR, micro RNA; siRNA, small interfering RNA; shRNA, short hairpin RNA; qRT-PCR, quantitative reverse transcription-polymerase chain reaction; WB, western blot; Seq, sequencing; HDACi, histone deacetylase inhibitors; WT, wild type; KO, knock out; α-Syn, α-synuclein; IRES, internal ribosome entry site; GFP, green fluorescent protein; ROS, reactive oxygen species; (s)PD, (sporadic) Parkinson’s disease; SNP, single nucleotide polymorphism.*

#### PD-Risk SNPs Are Enriched in Enhancers in DAn From LUHMES Cells

Pierce et al. (2018) evaluated genome-wide histone H3K27 acetylation, CCCTC-binding factor (CTCF) occupancy, and RNA expression in differentiated (6–7 days old) and undifferentiated LUHMES cells (Table 1). The differentiation into DAn was accompanied by distinct changes in gene expression. 57,905 genes were mapped in total and 6,147 genes were significantly downregulated whereas 7,621 genes were significantly up-regulated. Chip-Seq revealed 25,000 active enhancers, 12,000 active promoters, and 40,000 occupied CTCF sites between differentiated and undifferentiated LUHMES cells. PD-risk SNPs were enriched in enhancers for synapse and vesicle trafficking categories in DAn from LUHMES. However, the fold-change to enhancer location explained only 10% of variability in their data set. The authors suggested that enhancers do not only control the expression of single genes, but many genes are affected by particular loci.

#### MIR-7 Is Cytoprotective and Exhibits Anti-Neurotoxic Effects in ReNcells

MIR are small, highly conserved, sequence-specific non-coding RNA molecules regulating post-transcriptional gene expression (Sato et al., 2011) (Table 1). Concerning MIR alterations, the group of E. Junn showed that lentiviral overexpressed MIR-7 promoted glycolysis via downregulation of RelA in 2 weeks old ReNcell (human ventral mesencephalic neural progenitor)-derived DAn (Chaudhuri et al., 2015). MIR-7 is highly expressed in the brain, including DAn from the Substantia nigra. In their cell model, MIR-7 also increased the expression of the Glucose Transporter Glut3 and finally resulted in cytoprotection and exhibition of anti-neurotoxic effect after treatment with MPP^+^. The authors pointed out that an intact glycolytic pathway is necessary for neuronal survival and promoting effects of MIR-7 could be beneficial in PD. In a subsequent study, Choi et al. (2018) demonstrated that overexpression of MIR-7 in ReNcells resulted in a decrease of monomeric and high molecular weight forms of α-synuclein (Table 1). Interestingly, α-synuclein aggregates due to transfection of preformed α-synuclein fibrils were also diminished. The authors could show that an increased autophagic reflux promoting α-synuclein clearance was responsible for the observed effects.

#### MIR-221 Expression Is Modulated by WT DJ1 to Execute Cytoprotective Effects

DJ1 (PARK7) mutations lead to fPD (Bonifati et al., 2003). Thus, Oh et al. (2018) used an array-based screen to identify RNA transcripts regulated by DJ1 (Table 1). Using SH-SY5Y cells, they demonstrated that MIR-221 exhibited a 55% reduction of expression in DJ1 knock out (KO) cells and confirmed these findings via qRT-PCR as well as using a DJ1 KO mouse model. Solely the WT DJ1 protein seems to influence MIR-221 expression levels through the MAPK/ERK pathway. They further consolidated these findings in ReNcell VM showing that lentiviral transfection of MIR-221 was cytoprotective in DJ1 KO cells.

Overall, these studies demonstrated various epigenetic PD-associated changes in fetal human neurons. Their impact on PD pathogenesis requires further studies, especially using patient-specific neurons derived from aged individuals.

### Epigenetic Studies in iPSC-Derived Neurons From PD Patients

#### Aberrant DNA Methylation Profiles in iPSC-Derived DAn From PD Patients

Fernandez-Santiago et al. (2015) demonstrated that iPSC-derived DAn from PD patients exhibit an aberrant DNA methylation pattern compared to controls (Table 2). They differentiated fibroblast-derived iPSC into 30 days old dopaminergic neurons of the A9 subtype. In total, they generated DAn from four PD patients carrying *LRRK2* mutations (G2019S, L2PD), six sPD patients and four controls. The expression of DAn markers ranged from 44 to 67%. Applying array-based analysis for DNA methylation (450K, Illumina) and gene expression (Genechip Human Exon 1.0 ST, Affymetrix) this study demonstrated that L2PD and sPD exhibit similar DNA methylation and gene expression profiles. DAn from L2PD and sPD patients showed an aberrant DNA methylation and gene expression profile compared to fibroblasts, iPSC and non-dopaminergic neurons from PD patients and controls. DNA methylation alterations in DAn were enriched in enhancer elements, transcription factor (TF) binding sites and were partially correlated with gene expression alterations validated via qRT-PCR. Upregulated TFs and proteins included e.g., *OTX2/PAX6* or *SNCA/DCC*. The authors noted that DAn from PD patients might have undergone incomplete epigenomic remodeling compared to controls. The study showed an aberrant DNA methylation profile in DAn only – not other non-dopaminergic phenotypes - and very similar DNA methylations differences in DAn derived from the fPD and sPD cases. It is quite remarkable that familial and idiopathic PD cases show common epigenetic modifications in the most vulnerable neuronal subtype affected in PD.

**Table 2:** Epigenetic studies in human iPSC-derived neurons.

Study	Cell source	Neuronal differentiation	Number individuals	Methods	Results
Fernandez-Santiago et al., 2015	Fibroblast-derived iPSC	DAn, 30 days old; non-DAn, 30 days old	4 PD *LRRK2*, 6 sPD, 4 controls	450K Illumina, Gene-chip Human Exon 1.0 ST Affymetrix, qRT-PCR, WB	DAn from PD patients exhibit an aberrant DNA methylation pattern
Straniero et al., 2017	Fibroblast-derived iPSC	DAn, 35 days old	4 PD heterozygous*GBA* mutations (p.L444P *n* = 2, p.N370S *n* = 2), 6 controls	Semi-quantitative RT-PCR, Luciferase assays, WB, GCase activity assays	MIR-22-3p is downregulated in iPSC-derived DAn from PD patients carrying *GBA* mutations
Tolosa et al., 2018	Fibroblast-derived iPSC	DAn, 30 days old	3 PD *LRRK2*, 3 sPD, 4 controls	TaqMan Array human MicroRNA A cards 2.0, qRT-PCR, computational analysis	Altered MIR profiles in iPSC-derived DAn from PD patients
Tagliafierro et al., 2018	Fibroblast-derived iPSC	DAn forebrain cholinergic neurons, 45–50 days old	1 PD triplication patient, 1 control	Computational analysis, qRT-PCR, Immuncytochemistry, Sanger sequencing	Identification of 4 conserved MIR binding sites at the*SNCA* 3′ UTR
Gonzalez-Cano et al., 2018	Fibroblast-derived iPSC	Neuronal epithelial cells, 2 days old	n.a.	WB, IP, Luciferase assays, Immunostaining	TRIM23 antagonizes LRRK2 effect on MIR Let-7
Lang et al., 2018	Fibroblast-derived iPSC	DAn, 45 days old	4 sPD, 3 PD GBA-N370S, 3 controls	RNA-seq, computational assays, qRT-PCR, immunocytochemistry, WB, α-synuclein	HDAC4 activity and localization is perturbed in DAn from GBA and sPD patients
Kantor et al., 2018	Fibroblast-derived iPSC	DA neural progenitors, 21–23 days old	1 PD triplication patient	gRNA-dCAS9-DNMT3A lentiviral transfections, qRT-PCR, Immunocytochemistry, WB, mitochondrial superoxide and cell viability assays, MethylFlash Global DNA Methylation ELISA Kit	Gene edited increased DNA methylation levels of the*SNCA* intron 1 suppress gene expression and reverse PD-associated phenotypes

*DAn, dopaminergic neurons; n.a., not applicable; MIR, micro RNA; qRT-PCR, quantitative reverse transcription-polymerase chain reaction; WB, western blot; Seq, sequencing; HDAC, histone deacetylases; (s)PD, (sporadic) Parkinson’s disease; iPSC, induced pluripotent stem cells; LRRK2, leucine-rich repeat kinase; UTR, untranslated region; IP, immunoprecipitation; TRIM23, tripartite motif containing 23; GBA, glucosylceramidase beta; gRNA-dCAS9-DNMT3A, guide RNA-deactivated Cas9-DNA methyltransferase 3A.*

#### Altered MIR Profiles in iPSC-Derived DAn From PD Patients

Tolosa et al. identified MIR alterations in DAn from sPD and L2PD patients compared to controls (Tolosa et al., 2018) (Table 2). They used the very same samples as in the study of Fernandez Santiago et al. (Fernandez-Santiago et al., 2015). They first performed array-based and qPCR analyses to identify differentially expressed MIR. Different computational approaches were then applied to perform enrichment analysis and to compare the results with global gene expression data from the previous study. The functional analysis revealed two main clusters associated with proper neuronal (cluster 1) and diverse homeostatic (cluster 2) functions. Interestingly, the alterations were again identified in DAn from sPD and L2PD patients to a very similar extent. MIR alterations involved up- and downregulated MIR. These expression differences seem to display early molecular changes preceeding neuropathological cellular PD phenotypes such as inclusion formation.

#### TRIM23 Reverses Impaired LRRK2-Mediated MIR Let-7a Activity in Neuronal Epithelial Cells

Tripartite Motif Containing 23 (*TRIM23*) has been described as an activator of MIR-associated proteins and is supposed to regulate *SNCA* (Schwamborn et al., 2009; Pavlou et al., 2017). Gonzalez-Cano et al. (2018) showed that *LRRK2*, *TRIM23*, argonaute protein 2 and the MIR Let-7a interact *in vitro* and *in vivo* (Table 2). Vector-mediated expression of GFP-tagged TRIM23 and/or fly-tagged *LRRK2*-RR141H in neuronal epithelial cells (differentiated for 48 h) suggested that *LRRK2* inhibits MIR Let-7a resulting in impaired neuronal differentiation. These effects could be antagonized by *TRIM23* expression to some extent.

#### The SNCA 3′ UTR Contains Conserved MIR Binding Sites in iPSC-Derived Cholinergic Neurons

Tagliafierro et al. (2018) tried to identify conserved MIR binding sites at the 3′ UTR of *SNCA* (Table 2). They carried out a computational approach and further validated the MIR expression in iPSC-derived forebrain cholinergic neurons (45–50 days old) from a *SNCA* triplication patient and 1 control cell line. The *SNCA* 3′ UTR exhibited four conserved binding sites for MIR-7-5p, MIR-140-3p, MIR-153-3p, and MIR-223-3p. It has been previously shown that MIR-7 and MIR-153 regulate *SNCA* mRNA expression levels (Junn et al., 2009; Doxakis, 2010) and MIR-223 is involved in the differentiation of mature neurons (Harraz et al., 2014). However, the expression levels of all 4 MIR varied between iPSC, neural precursor cells and iPSC-derived neurons. Moreover, an association between genetic variants of the 3′ UTR and the conserved MIR binding sites could not be established.

#### MIR-22-3p Is Downregulated in iPSC-Derived DAn From PD Patients Carrying GBA Mutations

Straniero et al. (2017) focused on *GBA* expression regulation evaluating the effect of the MIR-22-3p and MIR-132 (Table 2). Preliminary analysis in HEK293 and HeLa cells demonstrated, that MIR-22-3p targets *GBA* and the *GBA* pseudogene *GBAP1*. These findings were validated in 35 days old iPSC-derived DAn from six controls and four PD patients carrying *GBA* mutations according to the differentiation protocol of Kriks et al. (2011). Upon neuronal differentiation, *GBA* and *GBAP1* were up-regulated and MIR-22-3p was downregulated in control DAn compared to the neuronal precursors. *GBA* transcripts were downregulated in iPSC-derived DAn from PD patients accompanied by a slight up-regulation of MIR-22-3p. Though a connection between the RNA-based network and PD pathology has not been firmly established, the results point toward a possible link between *GBA/GBAP1*and MIR-22-3p.

#### HDAC4 Mislocalization Leads to Protein Dyshomeostasis in iPSC-Derived DAn From GBA-N370S Patients

Another link between *GBA* mutations and epigenetic alterations was proposed by Lang et al. (2018) (Table 2). They observed an increase in nuclear localization of HDAC4 in iPSC-derived DAn from PD *GBA*-N370S patients compared to controls. Non-dopaminergic neurons did not exhibit this phenotype. The mislocalization of HDAC4 was associated with a downregulation of HDAC4-controlled genes. It is exciting that four compounds – used in other diseases such as prostate cancer – targeted HDAC4 activity, reversed the impaired expression of the HDAC4-controlled genes and subsequently ameliorated pertubations in autophagy and lysosomal pathways. Furthermore, these findings were also confirmed in four sPD cases.

#### DNA Methylation Editing of the SNCA Intron 1 Results in Gene Downregulation and Improvement of PD Phenotypes in iPSC-Derived Neuronal DA Progenitor Cell Lines

As already mentioned, the *SNCA* intron 1 might be a putative promoter region regulating *SNCA* expression (Jowaed et al., 2010). Thus, Kantor et al. (2018) generated guide RNA (gRNA)-deactivated Cas9 (dCas9)-*DNMT3A* lentiviral vectors and increased the DNA methylation levels at the *SNCA* intron 1 in neuronal DA progenitors from a patient with a *SNCA* triplication mutation (Table 2). The targeted DNA methylation editing resulted in decreased *SNCA* expression, reduced mRNA/protein levels and improved pathological phenotypes (superoxide production, cell viability) in the iPSC-derived neuronal DA progenitors. The transgenes increased the methylation levels across several CpGs of the *SNCA* intron 1 with minimal effects on global DNA methylation levels or expression of certain selected genes. CpG 6 and 7 of the *SNCA* intron 1 seemed to be major target sites. To the best of our knowledge, this is the first study showing mechanistic epigenetic analysis and their direct impact on gene expression being not only relevant for fPD but also sPD. We would like to point out that such studies indeed require human neurons and cannot be performed using post-mortem brain tissue.

## Perspectives

Human neuronal cells bear promising potential to study epigenetic alterations. Several studies already identified characteristic features with regard to DNA methylation, MIR and histone modifications in human neuronal *in vitro* PD models. These findings are quite encouraging and point toward possible epigenetic pathophysiological processes in PD. This will hopefully lead to the development of novel therapeutic strategies – however a definite confirmation of such findings *in vivo* and of re-juvenation awaits clarification.

Human iPSC-derived neurons offer several advantages over other cellular and *in vivo* models. They

• are patient-derived
• can be obtained in an unlimited amount
• offer the possibility to generate highly pure neuronal populations
• are available for co-culture studies or generating 3D brain (cerebral organoids) better recapitulating the native cerebral environment, as astrocytes and microglia are considered to be implicated in the pathogenesis of PD (Fellner et al., 2011; Booth et al., 2017; Subramaniam and Federoff, 2017)
• allow the direct investigation of the presumed effects of epigenetic alterations on gene expression
• allow high-throughput screening
• are accessible for gene editing

However, it is under intense scrutiny whether human neuronal cellular models are appropriate to study epigenetic modifications. Particularly, fetal neurons might not be suitable to study age-associated epigenetic patterns related to NDD. Concerning iPSC-derived neurons, three major caveats have to be addressed:

(1) The process of directed reprogramming itself is based on epigenetic modifications (Rando and Chang, 2012).

(2) Reprogramming resets the epigenetic pattern almost to zero (“epigenetic rejuvenation”), impairing studies of age-associated alterations (Rando and Chang, 2012).

(3) The epigenome of iPSC-derived neurons might differ substantially from native neurons.

These three issues have yet to be resolved. It has already been demonstrated, using isogenic human neural stem cell systems, that the reprogramming process itself does not lead to an aberrant DNA methylation or gene expression pattern (Choi et al., 2015; de Boni et al., 2018). To overcome the limitation of epigenetic rejuvenation, fibroblast-derived neurons were generated via direct conversion omitting a pluripotent state (Mertens et al., 2015). However, preservation of age-associated signatures was not replicated in a current study generating directly converted neurons without the use of Oct4 (Sheng et al., 2018). Another approach was proposed by introducing progerin, a truncated version of lamin A protein involved in Hutchinson–Gilford progeria syndrome (Miller et al., 2013). The authors suggested that the lack of a distinct neurodegenerative phenotype, especially neuronal protein inclusions, could be due to the age-reset during reprogramming (Miller et al., 2013). Indeed, the analysis of DAn grafts exhibiting progerin overexpression in mice demonstrated large multilamellar inclusions which are considered to be precursors of Lewy bodies (Miller et al., 2013).

However, a dysregulation of progerin expression has not been observed in PD. Moreover, it has been shown that the genetic background and differentiation protocols have a high impact on the epigenetic landscape in iPSC-derived neurons (de Boni et al., 2018). Comparing iPSC-derived forebrain neurons to native, post-mortem temporal lobe bulk brain tissue, it has been shown that upon neuronal differentiation, differences in actively transcribed genes, long intergenic non-coding RNAs (lincRNA), and DNA methylation patterns were diminished (Hjelm et al., 2013). IPSC-derived neurons differentiated for 140 days exhibited <25% gene expression, <16% lincRNA, and <30% DNA methylation differences compared to native brain tissue (Hjelm et al., 2013). Single gene analysis demonstrated variable gene expression patterns for neuronal, astrocytic, or oligodendroglial markers: some markers are remarkably similar to native brain tissue [e.g., microtubule-associated protein 2 (*MAP2*) or the serine/threonine protein kinase 6 (*PAK6*)], some increased [e.g., Tubulin Beta 3 Class III (*TUBB3*) or neurotrophic growth factor (*NGF*)], whereas astrocytic and oligodendroglial markers remained downregulated [e.g., oligodendrocyte transcription factor 2 (*Olig2*) or glial fibrillary acidic protein (*GFAP*)], possibly due to the optimization of *in vitro* cell culture conditions for neuronal differentiation (Hjelm et al., 2013). Moreover, iPSC-derived DAn exhibited substantially different gene expression patterns and an incorrect anterior–posterior axis patterning compared to native midbrain bulk tissue (Xia et al., 2016). Genes associated with immature DAn were highly expressed in iPSC-derived DAn (Xia et al., 2016). In this context, a comparison of iPSC-derived neurons to, e.g., FACS-sorted native neurons is required. Using native laser-capture microdissected or FACS-sorted neuronal populations (e.g., DAn and glutamatergic neurons), recent studies have begun to demonstrate profound epigenetic and transcriptomic differences between iPSC-derived neurons and native neurons (Montaño et al., 2013; Kozlenkov et al., 2014, 2015; Dong et al., 2018). However, the precise epigenetic and transcriptomic landscape of native neurons has yet to be determined and compared to iPSC-derived neurons.

## Conclusion

Human *in vitro* (patient-specific) neurons bear the potential to generate useful tools to study epigenetic modifications in PD. However, major efforts are still required to define the epigenetic landscape of these neurons compared to native neurons and determine age-associated epigenetic patterns. First of all, native (single cell) epigenomic genome-wide analyses, from, e.g., FACS-sorted brain tissue, have to be performed to define the variation of the normal epigenetic landscape of different neuronal and glial populations. Including cells from aged individuals, such analyses could be used to create a reference epigenome for further epigenetic evaluations using human (patient-specific) *in vitro* neurons. The epigenetic comparison of native and *in vitro* human neurons could then help to improve current differentiation protocols and might reveal whether observed epigenetic changes *in vitro* indeed correspond to a PD or other phenotypes.

## Author Contributions

All authors listed have made a substantial, direct and intellectual contribution to the work, and approved it for publication.

## Conflict of Interest Statement

The authors declare that the research was conducted in the absence of any commercial or financial relationships that could be construed as a potential conflict of interest.
